# Financial Toxicity in Renal Patients (FINTORE) Study: A Cross-Sectional Italian Study on Financial Burden in Kidney Disease—A Project Protocol

**DOI:** 10.3390/mps7020034

**Published:** 2024-04-14

**Authors:** Rossella Siligato, Guido Gembillo, Emanuele Di Simone, Alessio Di Maria, Simone Nicoletti, Laura Maria Scichilone, Matteo Capone, Francesca Maria Vinci, Marta Bondanelli, Cristina Malaventura, Alda Storari, Domenico Santoro, Marco Di Muzio, Sara Dionisi, Fabio Fabbian

**Affiliations:** 1 Nephrology Unit, University Hospital of Ferrara, 44121 Ferrara, Italy; rossella.siligato@ospfe.it (R.S.); alessio.dimaria@ospfe.it (A.D.M.); simone.nicoletti@ospfe.it (S.N.); laura.scichilone@ospfe.it (L.M.S.); matteo.capone@edu.unife.it (M.C.); francescamaria.vinci@edu.unife.it (F.M.V.); a.storari@ospfe.it (A.S.); 2 Department of Biomedical, Dental, Morphological and Functional Imaging Sciences, University of Messina, 98121 Messina, Italy; guidogembillo@live.it; 3 Unit of Nephrology and Dialysis, Department of Clinical and Experimental Medicine, University of Messina, 98121 Messina, Italy; domenico.santoro@unime.it; 4 Department of Clinical and Molecular Medicine, Sapienza University of Rome, 00189 Rome, Italy; emanuele.disimone@uniroma1.it (E.D.S.); marco.dimuzio@uniroma1.it (M.D.M.); 5 Department of Medical Sciences, University of Ferrara, 44121 Ferrara, Italy; marta.bondanelli@unife.it (M.B.); cristina.malaventura@unife.it (C.M.); 6 Nursing, Technical and Rehabilitation, Department DATeR Azienda Unità Sanitaria Locale di Bologna, 40121 Bologna, Italy; sara.dionisi@uniroma1.it

**Keywords:** financial toxicity, financial burden, social determinants of health, chronic kidney disease, dialysis

## Abstract

Financial toxicity (FT) refers to the negative impact of health-care costs on clinical conditions. In general, social determinants of health, especially poverty, socioenvironmental stressors, and psychological factors, are increasingly recognized as important determinants of non-communicable diseases, such as chronic kidney disease (CKD), and their consequences. We aim to investigate the prevalence of FT in patients at different stages of CKD treated in our universal health-care system and from pediatric nephrology, hemodialysis, peritoneal dialysis and renal transplantation clinics. FT will be assessed with the Patient-Reported Outcome for Fighting Financial Toxicity (PROFFIT) score, which was first developed by Italian oncologists. Our local ethics committee has approved the study. Our population sample will answer the sixteen questions of the PROFFIT questionnaire, seven of which are related to the outcome and nine the determinants of FT. Data will be analyzed in the pediatric and adult populations and by group stratification. We are confident that this study will raise awareness among health-care professionals of the high risk of adverse health outcomes in patients who have both kidney disease and high levels of FT. Strategies to reduce FT should be implemented to improve the standard of care for people with kidney disease and lead to truly patient-centered care.

## 1. Introduction

Social deprivation refers to the reduction of normal interaction between individuals and the rest of society. Social deprivation is a complex issue requiring a multifaceted approach that considers economic, educational, and mental health aspects. Physical and mental illness, poor education, and low socioeconomic status are correlated with deprivation. Social deprivation encompasses limited access to the social world due to low socioeconomic status, inadequate education and illness. Deprivation can reduce an individual’s freedom and cause social exclusion. Poverty, unemployment, lack of social support, exclusion from services and negative neighborhood attitudes lead to social exclusion. In a broad sense, societal perceptions can be naïve and judgmental in terms of assumptions about the relationship between socioeconomic deprivation and health. Household income, educational attainment, and type of employment can be measures of individual deprivation. Several factors increase the risk of financial distress, such as the wage-earner status of a diseased person, pre-illness-associated costs, the influence of and illness and its treatment on ability to work, type of national health-care assistance, household income, and the social stratum to which the person belongs. Concrete measures of financial distress include out-of-pocket medical costs, out-of-pocket costs as a percentage of income, reduction in income and assets, medical debt, trouble paying for necessities and medical bills, and psychological response to increased financial burden [1,2].

Deprivation and financial hardship are closely related concepts that refer to the lack of access to basic needs and resources due to limited financial means. While they share similarities, they have distinct characteristics. Deprivation encompasses the lack of essential goods, services, and opportunities necessary for a decent standard of living. It can include inadequate access to housing, health care, education, nutritious food, clean water, sanitation, transportation, and other fundamental needs. Deprivation is often assessed in terms of material conditions, and can have significant impacts on physical health, mental well-being, and overall quality of life. Financial hardship specifically refers to the difficulty or strain experienced by individuals or families due to financial constraints. It encompasses challenges such as inability to pay bills, afford necessary expenses, make ends meet, or maintain a certain standard of living. Financial hardship can result from various factors, including unemployment, underemployment, low wages, high living costs, unexpected expenses, medical bills, debt, and economic downturns. While deprivation and financial hardship are distinct concepts, they often intersect, as financial constraints can contribute to deprivation by limiting individuals’ ability to access essential goods and services. For example, individuals experiencing financial hardship may struggle to afford adequate housing, health care, or nutritious food, leading to deprivation in these areas.

A decade after a manuscript appeared in the literature describing the impact of cost on cancer patients’ experiences [3], this issue was increasingly recognized as critical to patients’ lives. A considerable body of published work is descriptive and demonstrates significant relevance to patient well-being and care. Financial toxicity (FT) is common and is reported in several cancer types, regardless of the choice of therapy [4]. FT refers to the economic burden or hardship that individuals may experience as a result of medical expenses and related costs associated with their health care. It is a term commonly used in the context of serious illnesses such as cancer, chronic conditions like kidney disease, and other long-term health issues. Several factors contribute to financial toxicity, including the following.

- Medical Expenses: This includes costs associated with doctor visits, hospital stays, surgeries, medications, medical equipment, and other health-care services.
- Insurance Coverage: Even with insurance, individuals may still face significant out-of-pocket costs in the form of deductibles, copayments, coinsurance, and uncovered services or treatments.
- Lost Income: Illness can lead to missed workdays, reduced work hours, or even the inability to work altogether, resulting in a loss of income for patients and their families.
- Caregiver Costs: Family members or friends who take on caregiving responsibilities may also experience financial strain due to the time and resources required to provide care.
- Travel and Lodging: Patients may need to travel long distances to access specialized care or treatment facilities, incurring additional expenses for transportation, lodging, and meals.
- Emotional and Psychological Costs: Dealing with the financial burden of illness can lead to stress, anxiety, depression, and other mental health issues for both patients and their caregivers.
- Impact on Quality of Life: Financial toxicity can force individuals to make difficult decisions about their health care, such as delaying or forgoing treatments, cutting back on essential expenses like food or housing, or even declaring bankruptcy.

To address financial toxicity, health-care providers, policymakers, and patient advocacy groups advocate for measures such as improved insurance coverage, increased access to affordable health-care services, transparency in pricing and billing, and support programs to assist patients with financial challenges. The profile of high-risk FT described in the literature consists of young, low-income, non-white, and female patients [5]. Even patients who survive after completing active treatment face FT, and it is associated with emotional distress, low quality of life, and increased physical symptom burden [4]. On the other hand, FT affects access to care, adherence to treatment, and participation in clinical trials [6], and influences patients’ decision-making processes and the patient–physician relationship. Evidence shows that patients like to talk about costs and how they can reduce the financial burden [7]. Hamel et al. studied videos of discussions about costs and duration thereof in patients suffering because of cancer. Discussion of costs was carried out in 45% of interactions between patients and oncologists. Patients started the discussion in 63% of cases and oncologists in 36%. However, conversation focused more frequently on the impact of the diagnosis on individuals’ opportunities rather than costs [8]. Better communication or financial navigation, eventually involving social services, could reduce the financial burden of price transparency [9,10].

Globally, society is aging, and the burden of age-related diseases has increased dramatically. Several chronic diseases contribute to excess health-care spending among older adults, especially when diagnosed with dementia [11]. Non-communicable diseases (NCDs) are the leading cause of death worldwide, and the average expenditure in public hospitals for NCDs is much higher than non-NCD households [12]. Tang et al. [13] examined the temporal trends of medical expenditure on hospitalization and the prevalence of catastrophic health expenditure (CHE) using more than 5,000,000 hospital records of older people living in a rural area in southeast China from 2010 to 2016. The number of CHE hospitalizations and the total number of hospitalizations more than doubled from 2010 to 2016. In 2023, Pei et al. showed that in China, the top three medical expenses are related to drug, consultation, and treatment fees [14]. We are conscious that Chinese data are different from those from Italy, one being that results derive from a rural population; however, considering this limitation, we can interpret Tang et al.’s [13] data as a danger signal for Western societies’ sustainability.

We must not forget that the course and duration of illness are important determinants of financial burden [15], with prolonged illness being more likely to be associated with financial burden. Conversely, financial hardship leads to an increased risk of multimorbidity [16]. Chronic diseases, including CKD, have become a common clinical problem for physicians and are recognized as a global public health problem [17]. In 2019, it was estimated that more than 850 million people worldwide suffer from kidney disease [18], with the overall prevalence increasing by 29.3% across all age groups. CKD is a common disease in those with low socioeconomic status [19], and socioeconomic disadvantage affects its progression [20]. Global mortality is affected by CKD both directly and indirectly, as it is an important risk factor for cardiovascular disease. In 2020, it was reported that CKD is the 12fth-leading cause of death worldwide and that its prevalence results in a loss of 35.8 million disability-adjusted life years (DALYs) and 25.3 million DALYs from cardiovascular disease can be attributed to kidney failure [21].

In a US study published in 2021 involving 1425 individuals suffering from CKD, representing approximately 2.1 million Americans, it was reported that 46.9% of subjects experienced financial hardship due to medical bills and 20.9% were unable to pay medical bills at all. The authors found that lack of insurance was the strongest determinant of financial distress [22]. Financial difficulties have also been reported in Italy. Perrone et al. conducted a multicenter prospective trial in people suffering lung, breast or ovarian cancer investigating the association of FT with clinical outcomes, including survival. At baseline, 26% of the 3670 investigated subjects reported FT, and the latter was significantly correlated with worse quality of life. During treatment, 22.5% developed FT that was significantly associated with an increased risk of death [23]. Several symptoms, including depression, fatigue, pain, and sexual dysfunction, have been reported to be more associated with changes in financial status in dialysis patients [24]. Depression, low self-esteem, feelings of shame, and stigmatization can form a vicious cycle in which financial hardship is both a cause and a result of mental health problems, ultimately leading to avoidance of health care.

Even if potential financial challenges are difficult topics to address, health-care professionals should raise them in clinical conversations [25]. The costs, especially out-of-pocket costs, are rarely made explicit, and the benefits of a clinical conversation about financial toxicity are difficult to quantify. On the other hand, FT causes health inequalities in access to and quality of care for various chronic conditions, but knowledge of this could help health professionals to improve the management of CKD patients. Assessment of FT prevalence in frailty-related diseases, including CKD, could support the efforts of health-care professionals. The latter should also be supported by policies that address the outstanding issues of access to care, exorbitant health-care costs, high out-of-pocket costs, and lack of paid sick leave to improve the effectiveness of care and close other important gaps in our social safety net [26].

## 2. Aims and Objectives

Patients with CKD should be prepared to pay a considerable amount of money out of pocket for prescriptions [27] because chronic disease management is expensive [28,29,30]. CKD is associated with poverty and other social determinants of health (SDoH), such as social environmental stressors and psychological factors [31]. Financial problems can be linked to physical health, mental health, satisfaction with social activities and relationships, and a declining quality of life [32]. A vicious circle could be hypothesized among the direct costs of care, the indirect impact on the patient’s ability to earn an income, and health [33]. Economic hardship affecting material conditions, psychological response to the lack of resources, and health-related coping behaviors are variables that should be studied to understand FT [34]. It has been suggested that the “comprehensive score for financial toxicity” (COST), developed and validated using 11 items, can reveal the increasing influence of socioeconomic conditions on cancer patients’ outcomes [35,36]. However, this score has not been validated in Italy, and a different diagnostic mean has been proposed for Italian oncology patients. That score is called the Patient-Reported Outcome for Fighting Financial Toxicity (PROFFIT) and consists of 16 items. It is currently the first score to assess FT in a country with a fully public health-care system [37]. Our aim is to describe FT in Italian CKD patients using the PROFFIT questionnaire.

## 3. Research Questions

Data on FT in Italian people with CKD do not exist, although people with kidney illness deal with a long-term condition. Over such a long period of time, things can change, especially from an economic point of view. Therefore, examining the prevalence and impact of FT in a long-term chronic disease in a fully public health-care system could improve the knowledge in the field of social determinants on health in a Western country like Italy. Moreover, the social composition of Italian society is changing due to migration flows from Africa and Asia of poor people. The National Health Service was established in 1978 to provide health care to all citizens and residents through a mixed public–private system under the control of the Ministry of Health and administered on a regional basis. Surgical procedures and hospital stays are completely free for everyone, regardless of their income. Prescription drugs can only be purchased if they have been prescribed by a doctor. Drugs are subsidized and require only a co-payment depending on the type of drug, as well as visits to specialists or diagnostic tests that require only a co-payment, and are free for the low-income class. The increasing number of people with economic problems could impact the stability of the Italian health system and the necessity of out-of-pocket medical costs.

## 4. Methodology

Subjects who wish to participate in this study will complete the PROFFIT questionnaire [37]. In the survey, 7 questions relate to the outcomes and 9 the determinants of FT (details in Table 1). Each question addresses the topics of monthly expenses, financial resources, concerns about the economic future, financial impact on medical care, leisure activities or obtaining essential goods, and ability to work. The determinants examined are the need for and costs related to commute to treatment, additional medical expenses and support from health-care professionals. The PROFFIT questionnaire has been recently validated [38].

The possible answers to each question are the following: “very much agree”, “agree substantially”, “agree partially”, and “do not agree at all.” Administration of the questionnaire could happen either as a paper document or as a digital version on tablet, according to the facilities at each center and the patient’s or parent’s choice. The second step of the study will be monitoring the conditions and collecting sociodemographic data.

### 4.1. Design

**Figure 1:**
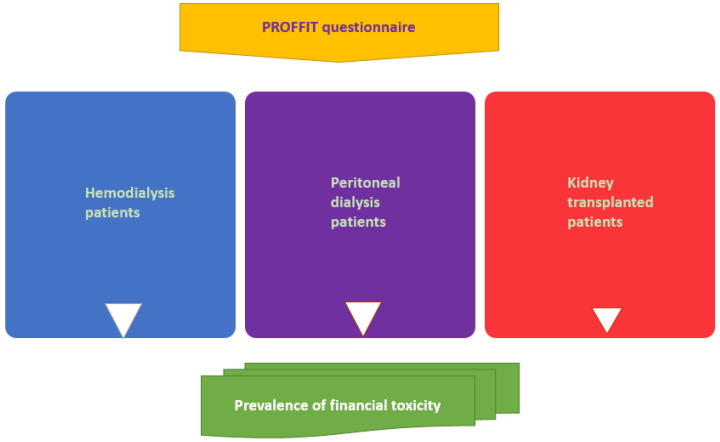
Conceptual framework of the study regarding the adults.

The design of the study is cross-sectional (Figure 1); however, we intend to keep on following up our population for at least 3 years in order to detect any variation in FT due to health-care system evolution and general dynamics of economy influencing inflation, income, and power of purchase. Our population of CKD patients will be recruited from hemodialysis, peritoneal dialysis and transplant outpatient clinics. Patients will be asked for their consent to participate anonymously in this study by completing the questionnaire. For pediatric nephrology patients, parents must give their consent before completing the PROFFIT questionnaire. All data will be stored in an electronic database.

### 4.2. Setting

Four different outpatient clinics that treat CKD at different stages will be involved. The Pediatric Nephrology Clinic treats patients with anomalies of the urinary tract, including congenital anomalies of the kidney and urinary tract (CAKUT). In this clinic, patients are regularly treated by the same group of pediatricians. Hemodialysis, peritoneal dialysis and transplantation outpatient clinics are part of the nephrology departments involved.

### 4.3. Participants (Eligibility Criteria)

The questionnaire will be administered to subjects who are willing to participate, have given their informed consent, and have been attending the pediatric nephrology, hemodialysis, peritoneal dialysis and transplantation clinics for at least six months. We will include patients affected by different stages of CKD.

All patients who do not give informed consent or refuse to participate in the study due to mental or cognitive disorders will be excluded. In addition, the 6-month follow-up requirement excludes patients with short-term kidney disease and/or patients with an acute complication requiring hospitalization and nephrology follow-up from this study.

Sample size calculation has been carried out using the tool freely available at https://clincalc.com/stats/samplesize.aspx (accessed on 20 December 2023). We have considered that in the general population, the prevalence of financial need for material, psychological and behavioral domains is around 20–50% for adults aged 18–64 years and around 15–30% for adults aged ≥65 years [39]. Moreover, in previous studies on CKD patients, FT has a prevalence between 30% and 70% [22,40,41,42,43,44]. Assuming that we are studying a population of subjects with CKD and that the primary endpoint is dichotomous (presence or absence of FT), we calculated an appropriate number of 137 patients when considering the lower limit of prevalence reported in the literature and 47 subjects when considering the upper limit of prevalence in the general and CKD population, respectively, taking into account a type I error of alpha 0.05 and a power of 80%.

On the other hand, this study being multicentric, we plan to enroll dialysis and kidney transplant recipients in different hospitals from the north and south of Italy. Dialysis patients will be engaged in the hemodialysis and peritoneal dialysis units and kidney transplant recipients in the specialized clinics. The reasons for participation in this study will be discussed with patients by trained nephrologists well known by them. Our aim is to enroll as many individuals as possible from different geographical areas of Italy in order to allow geographical comparison. We think that a total of 400 individuals will permit subgroup analyses.

### 4.4. Ethical Considerations, Consent, and Permission

The project will be conducted in accordance with the Declaration of Helsinki and the guidelines of good clinical practice. The local ethics committee of the Italian region of Emilia Romagna has given its approval for the study (650/2023/Oss/AOUFe). The document informing participants about the study contains the reasons for completing the questionnaire and the potential benefits arising from analyzing the results for both adults and parents of subjects under the age of 18. The research will not affect patients’ treatments. They will be informed that participation in the study is completely voluntary and that they can withdraw from the study at any time without negative consequences. The answers to the questionnaires will be treated confidentially and anonymously, and the handling of the data will also be confidential. The members of the research team will hand out the consent forms to the participants and will be available to answer any questions they may have about the study.

### 4.5. Data Management and Analysis

Given that the type of costs and determinants of FT will be different in children and adults, the pediatric population will be analyzed separately. Descriptive statistics will be used to report characteristics of demographic data. Continuous variables will be reported with median values and interquartile range (IQR) or mean values and standard deviation (SD), depending on the distribution of the variables evaluated with the Kolmogorov–Smirnov test. Categorical variables will be reported as absolute numbers and percentages. Compliance will be reported for the PROFFIT questionnaire. Data will be analyzed in the whole group and in the different groups of patients treated in the four different clinics to detect possible differences. Cronbach’s alpha will be calculated to assess the reliability of the questionnaire, given the previous use in a population different from people suffering kidney disease. Further statistical analyses will be performed using the chi-squared test to detect frequency differences and Student’s *t*-test and Mann–Whitney test to detect differences between normally distributed and abnormally distributed data, respectively. In addition, subgroup analysis by sex, age, ethnicity, and sociodemographic data will be carried out.

## 5. Expected Results

There are no data on the association between NCDs and FT in Italy. The results of our study will make it possible to compare prevalence data on FT from a fully public health system such as the Italian one with different health organizations. Our results will raise awareness of FT among health-care professionals. Only in this way can people with kidney disease and FT be considered at risk of negative health outcomes, leading to patient-centered care.

FT lacks a formal definition. FT describes financial problems of patients related to the medical management of their disease. Several costs related to a specific disease are not covered by health insurance or national health-care systems and may cause financial problems due to the increase of the cost of living. For this reason, FT affects a patient’s quality of life and access to medical care. Patients might not take prescription medicine or avoid going to see a doctor in order to save money. In order to understand FT, it is necessary to improve the understanding of it. The first step is to detect FT in certain subgroups of the population belonging to different countries with different organizations of the national health-care system. Sociodemographic characteristics such as age, race, marital status, education, geographic location and comorbidity have been associated with FT [45]. Also, travel and lodging costs should be taken into consideration. Patterns of FT can vary among different counties, and the presence of a public health insurance system is not always protective against FT [46]. In Japan, older age and higher household savings are positively associated with COST score, whilst nonregular employment, retirement because of cancer, and use of strategies to cope with the cost of cancer care are negatively associated with COST score, with lower COST scores indicating more severe FT [46]. Different tools have been suggested for measuring FT. Witte et al. performed a systematic review analyzing all detected instruments, items and questions with regard to their wording, scales and the domains of FT. They evaluated 41 publications based on 40 studies and highlighted six relevant items expressing perceptions of and reactions to FT: expenditure, exhaustion of financial resources, psychosocial responses, looking for support, handling care, and dealing with lifestyle changes. They detected a conflicting use of the six domains, making it difficult to compare and quantify the prevalence of FT. Moreover, not all instruments adapt to patients in third-party payer systems [47].

The COST score is a validated instrument to assess FT in patients from the United States [35,36]. COST correlates with health-related quality of life and provides a measurement of FT, although more research is necessary in order to study its relationship with outcomes. In Italy, the only validated means for detecting FT is the PROFFIT score, and clinical data are even more scanty [37]. Therefore, the efficacy of detecting FT with this instrument is still a matter of debate. More research is necessary to cover all relevant aspects of patients’ distress perception in order to engage health-care stakeholders. By engaging health-care stakeholders, it will be possible to involve policymakers to reinforce awareness of FT and initiate appropriate policy-level actions. All these steps could lead to a pursuit of strategies aimed at solutions beginning from clinical research. FT is a real clinical problem leading to adverse outcomes; therefore, measurement thereof allows for its recognition and discussion. Health-care professionals should be at the forefront, because recognizing and assessing FT means evaluating the development of future adverse outcomes. We believe that health-care professionals should incorporate social risk factors into health-care delivery and their medical decision-making processes in order to realize the best treatment able to improve patient outcomes.

## 6. Limitations

The main limitation of our study is the cross-sectional design, which is based on interviews with a limited sample of CKD patients. On the other hand, we plan to follow up our population to detect any variation in FT due to health-care system evolution and general dynamics of the economy influencing inflation, income, and power of purchase. The questionnaire does not take into account several parameters that could influence FT, including household income. However, we plan to collect sociodemographic data during the study’s follow-up. A survey able to investigate the existence of FT in a fully public health system such as the Italian one is only the first step in the knowledge of a very impactful problem. Detecting the reasons for the development of FT will be the subsequent step, and for carrying out this analysis, we should obtain sociodemographic data. Our results could set the stage for planning a survey involving a larger population and including analysis of the different risk factors for the development of FT.

Moreover, our study does not focus on a specific subpopulation, but includes subjects attending different clinics with different degrees of CKD, each requiring specific treatment. Obviously, focusing on a specific population can significantly help to focus attention on a centered care plan to personalize the treatment of these patients, also taking into account the aging of the population. Certainly, age plays a crucial role, as does income, so future studies should take these two important parameters into account.

## 7. Future Direction

Awareness of the problem and associated risk factors will encourage the planning of strategies to reduce the impact of FT on patients with non-communicable diseases, such as kidney disease, to improve their care and rationalize treatment by adapting it to clinical conditions. Guidelines on these matters are desirable.

## Figures and Tables

**Table 1:** The PROFFIT (Patient-Reported Outcome for Fighting Financial Toxicity) questionnaire.

	Outcome items
1.	I can afford my monthly expenses without difficulty.
2.	My illness has reduced my financial resources.
3.	I am concerned by the economic problems I may have in the future due to my illness.
4.	My economic situation affects the possibility of receiving medical care.
5.	I have reduced my spending on leisure activities such as holidays, restaurants or entertainment in order to cope with expenses related to my illness.
6.	I have reduced spending on essential goods in order to cope with expenses related to my illness.
7.	I am worried that I will not be able to work due to my illness.
	Determinant items
8.	The National Health Service covers all health costs related to my illness.
9.	I have paid for one or more private medical examinations for my illness.
10.	I have paid for additional medicines or supplements related to my illness.
11.	I have to pay for additional treatment myself (e.g., physiotherapy, psychotherapy, dental care).
12.	The treatment center is a long way from where I live.
13.	I have spent a considerable amount of money on travel for treatment.
14.	Medical staff (i.e., doctors, nurses) have been helpful throughout my medical care.
15.	Staff in hospital administration (i.e., for booking appointments, secretaries) have been helpful throughout my medical care.
16.	Medical staff and medical facilities I attended communicated with each other.
